# Research trends in pediatric diseases in North Korea: a scoping review of North Korean medical journals, 2006–2019

**DOI:** 10.1186/s13690-025-01547-x

**Published:** 2025-03-21

**Authors:** I Re Lee, Hae Won Lee, Hanna Jung, Songyi Han, Shinki An, Woo Taek Jeon

**Affiliations:** 1 Department of Medical Education, Yonsei University College of Medicine, Seoul, Republic of Korea; 2 Department of Pediatrics, Yonsei University College of Medicine, Seoul, Republic of Korea; 3 Department of Pediatrics, Hallym Hospital, Incheon, Republic of Korea; 4 Department of Psychiatry, Yonsei University College of Medicine, Seoul, Republic of Korea

**Keywords:** North Korea, Research trends, Pediatric research, Medical journal

## Abstract

**Background:**

This study analyzed the status of research trends in pediatric diseases in North Korea, as reported in North Korean medical journals. Despite the censorship and control exerted by the North Korean government, these journals provide critical insights into practical achievements and disease cases, serving as vital data sources for understanding North Korea’s health landscape.

**Methods:**

We reviewed 10 North Korean medical journals published from 2006 to 2019, specifically targeting the journals Pediatrics, Obstetrics and Gynecology; Surgery; and Chosun Medicine. From an initial pool of 7,739 articles, 728 pediatric research articles were selected for in-depth analysis. We categorized the articles according to publication year, field, article type, research method, statistical approach, and content. Pediatric diseases were classified using the International Classification of Diseases, Tenth Revision. We additionally investigated temporal changes, particularly between the Kim Jong-il and Kim Jong-un eras.

**Results:**

Our analysis of pediatric research articles from North Korean medical journals demonstrated a consistent format and brevity, with the frequent inclusion of the ruling authority’s directives and a lack of ethical discussion. Notably, epidemiological studies and randomized controlled trials were not reported. The research focused instead on practical applications, addressing high-burden diseases and the therapeutic effects of traditional medicines. Our distribution analysis revealed that congenital malformations (16.2%), infectious diseases (12.6%), respiratory diseases (11.2%), and gastrointestinal diseases (10.9%) were the most frequently studied topics, reflecting the primary causes of pediatric mortality in North Korea. A significant increase in the volume and diversity of pediatric research was observed during the Kim Jong-un era, coinciding with improved economic conditions and an increased emphasis on science and technology policies.

**Conclusions:**

North Korean pediatric research exhibits distinct and practical characteristics, with trends of increasing diversity and volume over time. Our findings highlight the ongoing autonomous development of pediatric medicine in North Korea, which could have positive implications for future pediatric research in the country. Understanding these research trends is essential for developing strategies to improve children’s health in North Korea.

**Table:** 

Text box 1. Contributions to the literature	
North Korean medical journals offer a rare perspective on pediatric health issues, addressing significant gaps in global health data from an isolated country. Understanding the trends and characteristics of pediatric research in North Korea can help policymakers and healthcare practitioners develop targeted interventions. Such insights can help improve pediatric health outcomes by focusing on prevalent diseases and aligning interventions with the unique health challenges faced by North Korean children. This study also contributes to the broader global health discussion by shedding light on healthcare in politically isolated regions.	

## Introduction

Even in the twenty-first century, North Korea has preserved its enigmatic status, perpetuating a hereditary dictatorship over three generations while maintaining its communist ideology. The regime has exercised stringent control over the inflow and outflow of information, selectively releasing data that often cannot be independently verified. This limited and filtered dissemination has hindered the global community’s capacity to understand the true state of affairs in the region, especially since the division of the Korean Peninsula. As such, the global community’s perspective on North Korea is mainly based on selectively shared information or accounts from defectors and foreign workers within the country.

International attention to North Korea has intensified since the severe famine of the 1990s, which reportedly resulted in about 600,000 deaths due to economic hardship between 1995 and 2000 [1]. Although there have been indications of some improvements in food availability under Kim Jong-un’s rule, chronic shortages persist, with the World Health Organization (WHO) estimating that 10.9 million citizens (42.4% of the population) suffered from malnutrition between 2018 and 2020 [2]. Such widespread malnutrition has severe implications, particularly for children, affecting their long-term health and increasing mortality and morbidity rates [3].

Analysing North Korean citizens’ current health status requires relying on reports from South Korea’s Statistics Korea, the United Nations Children’s Fund (UNICEF), the WHO, and the United Nations. According to the ‘State of the World’s Children 2021’ report published by UNICEF, in 2020, the under-five mortality rate in North Korea was 17 deaths per 1,000 live births—six times higher than that of South Korea [4]. However, data related to the specific diseases leading to this high mortality rate are unavailable. Although Statistics Korea provides data on child health (including infant mortality), mortality rates for children under five years of age, malnutrition, and maternal mortality rates, it lacks data on specific pediatric diseases. Existing reports on the nutritional status and growth of children in North Korea are available, but it is difficult to find studies that report statistics related to pediatric diseases [5, 6].

Regarding the information we need to understand the specific diseases affecting North Korean children, the only publicly accessible information is found in quarterly North Korean medical journals. These publications can be accessed through the Information Center on North Korea, run by the South Korean Ministry of Unification. Despite the inability to obtain journals published after 2019, there are over 60 scientific periodicals published in North Korea, 10 of which are medical journals [7]. Unlike typical medical journals, those published in North Korea fall under the jurisdiction of the Propaganda and Agitation Department of the North Korean government and are subject to censorship. Additionally, the selection of researchers for submission and publication is subject to interference from the North Korean government [8]. Despite these functional limitations, these North Korean medical journals publish information related to outcomes in specific academic fields and research conducted on case reports of diseases, thus highlighting their importance for understanding the closed-off status of pediatric disease in North Korea.

Furthermore, consistent access to published data is necessary to predict changes in the health environment and disease patterns over time. North Korean medical journals began publishing in 1954 and have since been published quarterly in various fields. Thus, North Korean medical journals can be considered appropriate data sources for analysing trends in pediatric scientific research and changes in the prevalence of pediatric diseases across different eras in North Korea. Several studies in various medical fields have been published to predict North Korean conditions based on these North Korean academic medical journals [9–14].

In light of the above, this study aimed to extract, classify, and analyze North Korean medical journal articles related to pediatrics to examine the characteristics and trends of pediatric research in the country and understand changes over time. In doing so, this study seeks to establish a foundational framework for future investigations of North Korean children’s health.

## Methods

### Research design

We conducted a scoping review to extract and analyze pediatrics-related articles from North Korean medical journals. The study was designed using the methodological framework proposed by Arksey and O’ Malley [15]. To enhance the research design, we applied the method outlined by Levac et al. during the analytical phase [16]. The research design comprised five stages.

#### Step 1: identifying the research questions

In the first step, we formulated the following research questions for this study:

1. General characteristics of research articles: What are the general characteristics (journal name, publication year, subfield, authors, article type, research methods, statistical methods, and content) of pediatrics-related research articles in North Korean medical journals?
2. Classification of pediatric diseases and treatments: How are the pediatric diseases and treatments studied in these journals classified? What aspects of the research content are emphasized and is there research on diseases known to cause significant mortality?
3. Changes in research trends over time: Have there been significant changes in the diseases studied and the content of the research?

#### Step 2: identifying relevant studies

We identified relevant studies in the second step. Currently, 10 North Korean medical journals (*Juche Medicine*; *Chosun Medicine*; *Medicine*; *Preventive Medicine*; *Basic Medicine*; *Internal Medicine*; *Dental-Ophthalmology-Otolaryngology*; *Koryo Medicine*; *Pediatrics*,* Obstetrics and Gynecology*; and *Surgery*) can be officially accessed in South Korea. We obtained North Korean medical journals from the Information Center on North Korea (https://unibook.unikorea.go.kr/), which belongs to the Ministry of Unification, Republic of Korea. Through preliminary investigation focusing on the titles of each journal’s articles, four North Korean medical journals containing pediatrics-related research articles were selected as analytical data: *Pediatrics*,* Obstetrics and Gynecology*; *Surgery*; *Chosun Medicine*; and *Medicine*. We excluded the remaining six journals because their main research fields were beyond the scope of this study. *Juche Medicine* and *Koryo Medicine* were excluded due to their emphasis on traditional medicine. *Preventive Medicine*,* Basic Medicine*, *Dental-Ophthalmology-Otolaryngology*, and *Internal Medicine* were excluded because they mainly focus on fields outside pediatrics or on adult-specific research. Among these, three journals—*Pediatrics*,* Obstetrics*,* and Gynecology*; *Surgery*; and *Chosun Medicine*—were selected based on their overlapping publication period (2006–2019) to capture consistent changes over time. *Medicine* was excluded because its publication years (1988–2008) did not align with the selected period, and including it would have disproportionately represented the Kim Jong-il era, potentially distorting the comparative analysis between the Kim Jong-il and Kim Jong-un eras.

To identify relevant studies, we employed a three-step search strategy based on the Joanna Briggs Institute protocol [17]. The initial identification was conducted based on the titles listed in the journals, followed by a secondary identification process by reviewing the references.

#### Step 3: study selection

We reviewed articles from the selected journals—*Pediatrics*,* Obstetrics and Gynecology; Surgery;* and *Chosun Medicine*. The selection process involved reviewing titles and abstracts to identify articles that presented research results in the field of pediatrics.

We also included studies targeting children and focusing on specific diseases. Our exclusion criteria included: studies lacking clear disease presentation or providing limited information, covering solely specific procedures or surgeries, and studies focusing on normal child growth and development, specific test results, or demographic statistics. The scope was limited to the medical field, excluding dentistry- and traditional medicine-related research. Original articles, case reports, and reviews were included, whereas editorials were excluded.

After the initial review, we reviewed the full texts of all of the selected articles and applied the exclusion criteria to determine the final sample. In the second stage, two researchers with extensive clinical experience in pediatrics independently reviewed the selected articles. The articles were classified according to the International Classification of Diseases, Tenth Revision (ICD-10). In cases where opinions differed, consensus was reached through discussion among the researchers.

#### Step 4: charting the data

To extract data from the reviewed articles, we selected analysis variables based on the research characteristics and outcome extraction tools outlined by the Joanna Briggs Institute [17]. Data were extracted based on the article title, journal name, publication year, research subfield, article type, research topic, research methods, statistical methods, research content, and treatment methods.

#### Step 5: collating, summarizing, and reporting the results

Using descriptive statistics, the general characteristics of the study participants were analyzed based on the extracted data. We conducted frequency analysis to examine the articles’ general characteristics, disease distribution, and research content. The data analysis results are presented as frequencies and percentages and categorized according to journal name, publication year, subfield, article type, research methods, statistical methods, disease classification of research topics, research content, and treatment methods. We also conducted trend analyses and statistical tests (linear-by-linear association and chi-square tests). All statistical analyses were performed using IBM SPSS version 25.0.

### Ethical considerations

This study analyzed articles published in North Korean medical journals. As it did not involve research on human subjects, it was not subject to review by an Institutional Review Board and did not require separate approval.

## Results

**Fig. 1:**
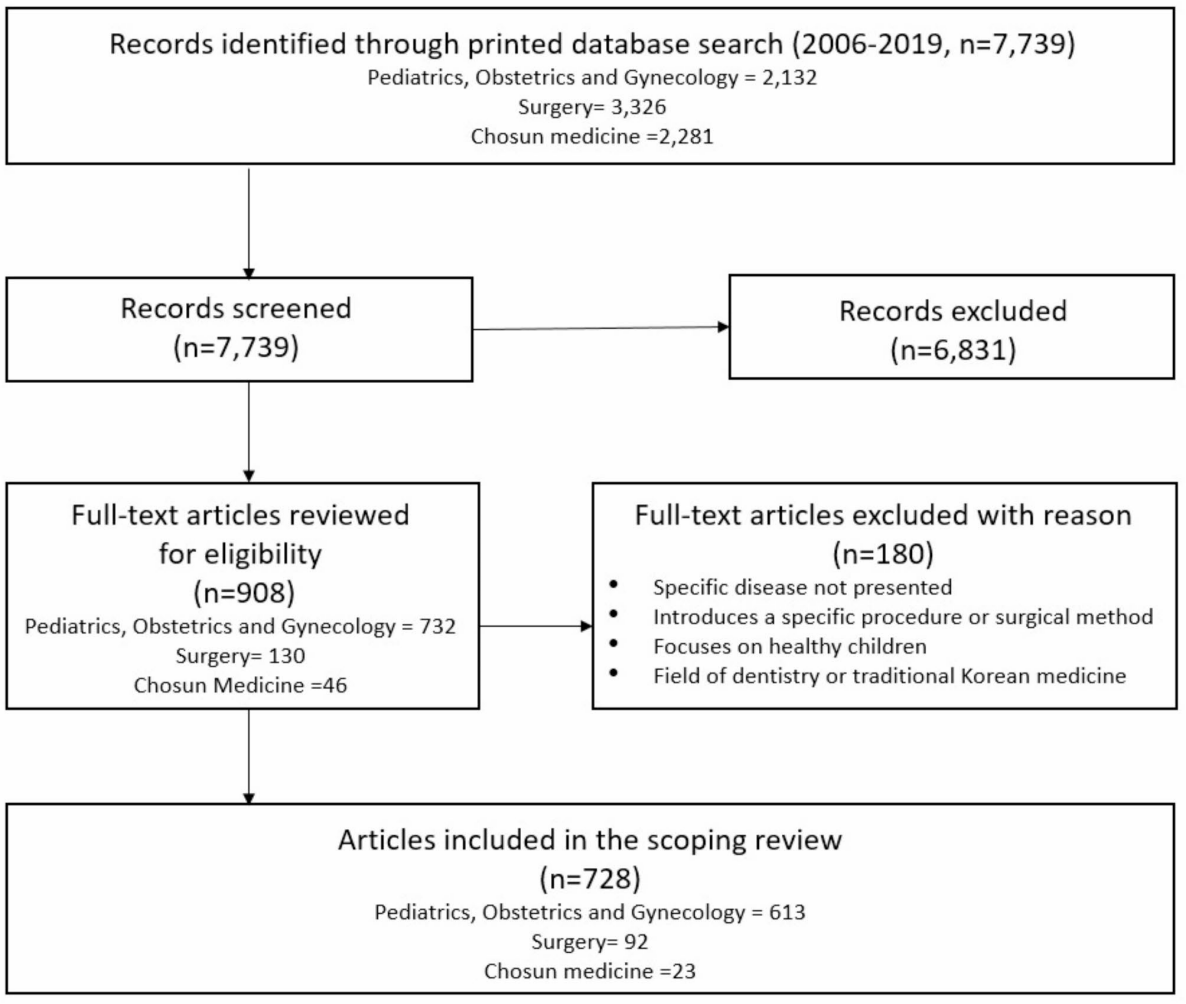
Flowchart of data collection and study selection processes in scoping review

The initial search yielded 7,739 articles published over 14 years, from 2006 to 2019, in the journals *Pediatrics*,* Obstetrics and Gynecology*; *Surgery*; and *Chosun Medicine.* After initial screening, 908 pediatrics-related articles were selected, including 732 from *Pediatrics*,* Obstetrics and Gynecology*; 130 from *Surgery*; and 46 from *Chosun Medicine*. After reviewing the full texts based on the inclusion and exclusion criteria, the final analysis targeted 728 articles, with 613 from *Pediatrics*,* Obstetrics and Gynecology*; 92 from *Surgery*; and 23 from *Chosun Medicine* (Fig. 1).

### General characteristics of the research articles

Among the analyzed North Korean medical journals publishing pediatrics-related research, the most prominent was *Pediatrics*,* Obstetrics and Gynecology*, accounting for 84.2% (613 of 728 articles) of all pediatrics-related articles.

The articles included the following sections: title, author information, research objectives, participants, methods, results, and conclusions. Most articles were less than two pages. The research objectives often mentioned the influence of the ruling authorities’ policies. None of the articles mentioned ethical considerations related to the research.

Regarding the types of research articles, original articles were the most prevalent (63.3%), followed by case reports (21.7%) and reviews (15.0%). Regarding methods, case-control studies were the most common, reported in 42.2% of the articles, followed by case series studies (28.7%) and case reports (25.5%). Experimental studies accounted for 3.4% of the total sample, with no randomized controlled trials conducted. In terms of statistical methods, basic descriptive statistics (e.g., means and frequencies) were most commonly found (50.9%), followed by t-tests to verify significant differences (37.8%), and chi-square tests for categorical data analysis (8.8%) (Table 1).

**Table 1:** Classification of research types, methods, statistical methods, and research contents

Research type	*N* (%)	
Original articles	461 (63.3)	
Review articles	109 (15.0)	
Case reports	158 (21.7)	
**Research method**	**N (% of 619)**	
Case-control study	261 (42.2)	
Case series	178 (28.7)	
Case report	158 (25.5)	
Experimental study	21 (3.4)	
Cohort study	1 (0.2)	
Randomized controlled trials	0 (0.0)	
**Statistical method**	N (% of 558*)	
Descriptive statistics	284 (50.9)	
Mean analysis	211 (37.8)	
Categorical data analysis	49 (8.8)	
Analysis of variance (ANOVA)	1 (0.2)	
Regression analysis	7 (1.3)	
Artificial neural networks	6 (1.1)	
**Research content**	N (% of 471*)	
Treatment effects	282 (59.9)	
Test results	80 (17.0)	
Clinical characteristics	57 (12.1)	
Diagnosis	30 (6.4)	
Pathogenesis, incidence, etiology	13 (2.8)	
Differential diagnosis	9 (1.9)	
Epidemiology	0 (0.0)	

Note: *Duplication allowed in the original article

We classified the studies based on full-text reviews of original articles on epidemiology, diagnosis, differential diagnosis, pathogenesis, incidence, etiology, clinical characteristics of disease, test results, and therapeutic effects. The majority of the articles (282; 59.9%) focused on therapeutic effects. Additionally, 80 articles (17.0%) focused on test results; 57 (12.1%) on clinical characteristics; 30 (6.4%) on diagnosis; 13 (2.8%) on pathogenesis, incidence, and etiology; and 9 (1.9%) on differential diagnosis. However, no epidemiological studies were conducted (Table 1).

The most active research subfields were pediatric gastroenterology and nutrition (23.9%), followed by pediatric allergy and respiratory diseases (11.1%), pediatric surgery (8.1%), pediatric cardiology (7.4%), and pediatric neurology (6.6%) (Table 2).

**Table 2:** Classification of detailed research subfields across ruling periods

Subfield		KJI era	KJU era	*N* (% of 728)
Pediatric Gastroenterology & Nutrition		69 (31.4)	105 (20.7)	174 (23.9)
Pediatric Allergy and Respiratory diseases		30 (13.6)	51 (10.0)	81 (11.1)
Pediatric Surgery		14 (6.4)	45 (8.9)	59 (8.1)
Pediatric Cardiology		15 (6.8)	39 (7.7)	54 (7.4)
Pediatric Neurology		12 (5.5)	36 (7.1)	48 (6.6)
Pediatric Orthopedics		8 (3.6)	36 (7.1)	44 (6.0)
Neonatology		17 (7.7)	25 (4.9)	42 (5.8)
Pediatric Nephrology		15 (6.8)	21 (4.1)	36 (4.9)
Pediatric Infectious Diseases		14 (6.4)	19 (3.7)	33 (4.5)
Pediatric Psychiatry		3 (1.4)	27 (5.3)	30 (4.1)
Genetics		0 (0.0)	24 (4.7)	24 (3.3)
Pediatric Dermatology		5 (2.3)	18 (3.5)	23 (3.2)
Pediatric Hematology & Oncology		9 (4.1)	12 (2.4)	21 (2.9)
Pediatric Endocrinology		0 (0.0)	15 (3.0)	15 (2.1)
Pediatric Urology		4 (1.8)	8 (1.6)	12 (1.6)
Pediatric Neurosurgery		1 (0.5)	10 (2.0)	11 (1.5)
Pediatric Rheumatology		2 (0.9)	8 (1.6)	10 (1.4)
Pediatric Otolaryngology		1 (0.5)	3 (0.6)	4 (0.5)
Pediatric Thoracic Surgery		1 (0.5)	3 (0.6)	4 (0.5)
Pediatric Anesthesiology		0 (0.0)	1 (0.2)	1 (0.1)
Pediatric Ophthalmology		0 (0.0)	1 (0.2)	1 (0.1)
Pediatric Rehabilitation		0 (0.0)	1 (0.2)	1 (0.1)
**Total**		220 (100.0)	508 (100.0)	728 (100.0)

Note: KJI Kim Jong-il, KJU Kim Jong-un

Our assessment of research quality based on the standards of the Scottish Intercollegiate Guidelines Network (SIGN) indicated that most of the studies were of low quality [18]. Many of the required criteria for high-quality research were not met or deemed non-evaluable.

### Classification of pediatric diseases and treatments

#### Distribution of pediatric diseases

**Fig. 2:**
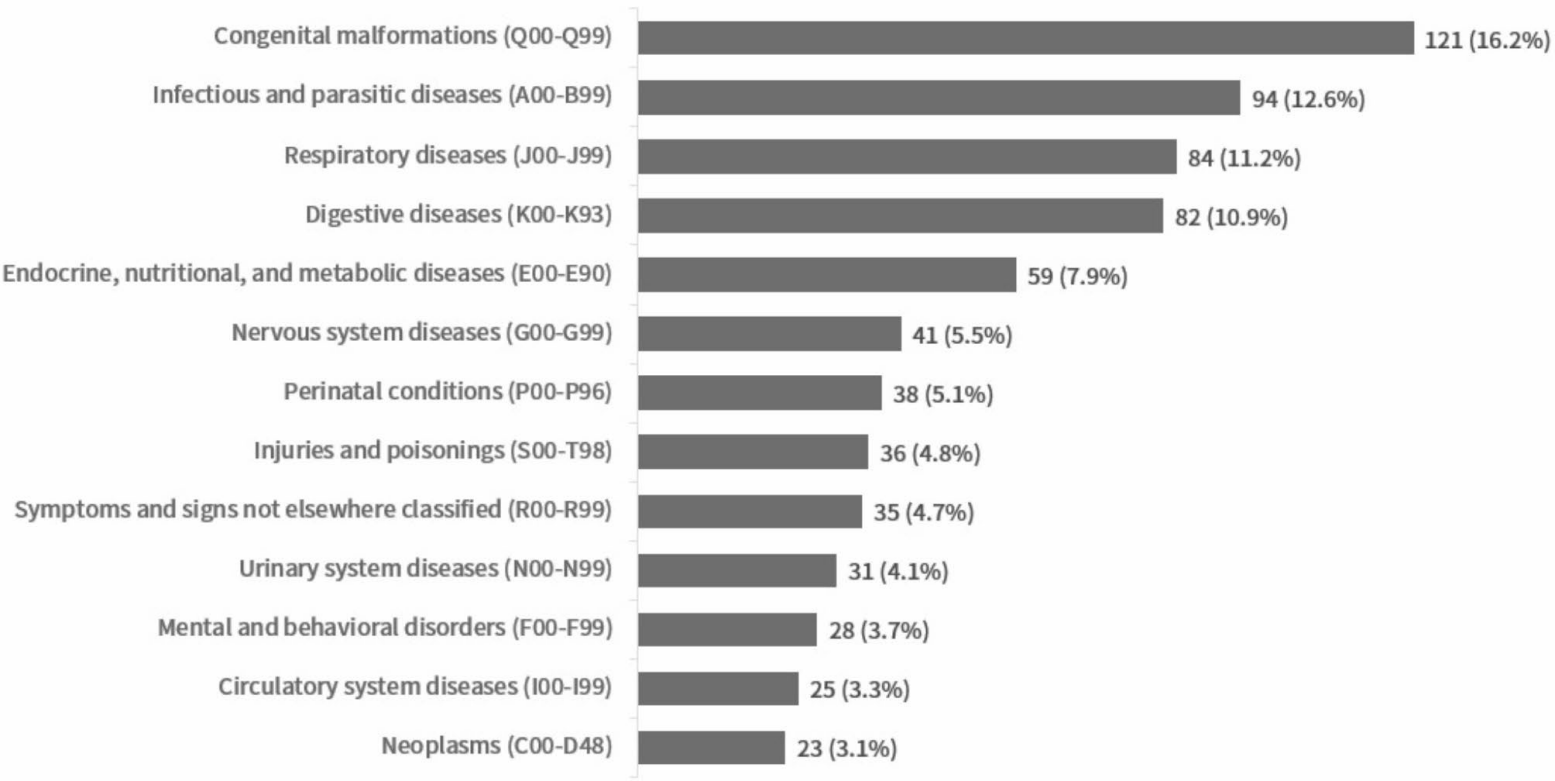
Distribution of pediatric diseases in research articles according to ICD-10 categories (*N* = 749)

We used the ICD-10 to classify pediatric diseases. The analysis of the 728 articles resulted in 749 topics, allowing for duplicate articles. The most frequently examined topic was related to congenital malformations (Q00-Q99), accounting for 16.2% (121 articles) of the articles. Next, topics related to infections and parasitic diseases (A00-B99) were published in 94 articles (12.6%), followed by respiratory diseases (84 articles; 11.2%), gastrointestinal diseases (82 articles; 10.9%), and endocrine, nutritional, and metabolic diseases (59 articles; 7.9%) (Fig. 2).

Our data revealed that 50.1% of the articles investigated the causes of mortality in children under the age of five years in North Korea; these included prematurity, acute respiratory infections, neonatal asphyxia, congenital anomalies, diarrhea, neonatal sepsis, and other significant infectious diseases such as meningitis (Table 3) [19].

**Table 3:** Frequency of articles related to causes of mortality in children under 5 years old, classified by ICD-10 codes

Cause	ICD code		*N* (%)	
Prematurity	P05.0, P07.3, P22.0, P91.6		11 (1.5)	
Acute respiratory infections	J00, J03.0, J06.9, J09, J15.7, J18.0, J18.9, J20.4, J20.9, J21.0, J22		60 (8.2)	
Neonatal asphyxia	P21.0, P29.0		10 (1.4)	
Congenital anomalies	Q00-Q99		121 (16.6)	
Diarrhea	A04.9, A08.0, A09.0, K52.1, K52.9		88 (12.1)	
Injuries	S00-T98		36 (4.9)	
Neonatal sepsis and infections	A07.2, A16.7, A18.8, B08.4, B15.9, B34, B37.0, B66.1, J31.0, K12.0, K12.1, K35.8, L04.0, P35.1, P36.9, P39.9		31 (4.3)	
Meningitis	A17.0		8 (1.1)	
**Total**			**365 (50.1)**	

#### Classification of treatment

Of the 282 articles on therapeutic effects, the most extensively studied therapeutic modality was traditional medicine (103 articles; 36.5%) (Table 4). Traditional medicines contain substances derived from plants, animals, and minerals. Research in this domain spanned 11 subfields, with the highest concentration being in pediatric gastroenterology and nutrition and pediatric allergy and respiratory diseases.

**Table 4:** Classification of treatments in research articles across ruling periods (*N* = 282)

Treatment method		KJI era	KJU era	*N* (%)
Traditional medicine		46 (46.0)	57 (31.3)	103 (36.5)
Traditional medicine alone		37 (37.0)	40 (22.0)	77 (27.3)
Combined with Western medicine		9 (9.0)	17 (9.3)	26 (9.2)
Western medicine		24 (24.0)	64 (35.2)	88 (31.2)
Surgical or procedural interventions		8 (8.0)	35 (19.2)	43 (15.2)
Nutritional supplements		11 (11.0)	15 (8.2)	26 (9.2)
Other therapeutic interventions		10 (10.0)	6 (3.3)	16 (5.7)
Rehabilitation therapies		1 (1.0)	5 (2.7)	6 (2.2)
**Total**		100 (100.0)	182 (100.0)	282 (100.0)

Note: *Chi-square test showed a significant difference in the proportion of articles on traditional and Western medicine across ruling periods* (*p* = *0.013*). KJI Kim Jong-il, KJU Kim Jong-un

Western medicine was the next most examined therapeutic approach (88 articles, 31.2%). Studies of Western medicine covered 15 subfields, indicating diverse research interests, compared with traditional medicine. The most studied subfields were pediatric allergy and respiratory diseases and pediatric gastroenterology and nutrition.

Surgery was the third most examined treatment method (43 articles; 15.2%). Articles focusing on surgical efficacy spanned eight subfields, indicating a narrower focus than pharmacotherapy. The most studied subfields were surgery and orthopedic surgery.

Twenty-six articles (9.2%) examined the efficacy of nutritional supplements, with 24 articles in the field of pediatric gastroenterology and nutrition and two in pediatric hematology-oncology. Studies of other therapies (16 articles) and rehabilitation treatments (six articles) comprised 22 articles (7.9%).

### Changes in research trends over time

#### Changes in the characteristics of the research articles over time

The number of pediatrics-related articles gradually increased from 2006 to 2019. Our analysis of the characteristics of the research articles, divided by the reign periods of Kim Jong-il (2006–2011) and Kim Jong-un (2012–2019), revealed that research articles were published more than twice as frequently during Kim Jong-un’s reign. Pediatric gastroenterology and nutrition, as well as pediatric allergies and respiratory diseases, were the most actively examined fields during both reigns. However, compared with the Kim Jong-il era (31.4% in pediatric gastroenterology and nutrition and 13.6% in pediatric allergy and respiratory diseases), the proportion of research in these two areas decreased significantly during the Kim Jong-un era (20.7% for pediatric gastroenterology and nutrition and 10.0% for pediatric allergy and respiratory diseases). Additionally, while research in areas such as neonatology (7.7%), pediatric cardiology, and nephrology (6.8%) followed the two aforementioned fields during the Kim Jong-il era, there was a shift toward pediatric surgery (8.9%), pediatric cardiology (7.7%), and pediatric neurology (7.1%) during the Kim Jong-un era (Table 2). A comparison of the domains of articles in terms of internal medicine and surgery revealed a significant increase in surgical domain articles during the Kim Jong-un era, compared with the Kim Jong-il era (*P* = 0.014, odds ratio [OR] = 1.613, 95% confidence interval [CI] = 1.101–2.364).

#### Changes in treatment over time

We examined changes in the use of traditional and Western medicine over time. Analysis using linear-by-linear association statistics revealed that the utilization ratio of Western medicine increased over time, compared with that of traditional medicine (*P* = 0.019). Furthermore, chi-square tests stratified by the reign periods of Kim Jong-il (2006–2011) and Kim Jong-un (2012–2019) showed a significantly lower frequency of traditional medicine usage during the Kim Jong-un era. This indicates a progressive decline in research frequency concerning traditional medicine relative to that of Western medicine in the Kim Jong-un era (*P* = 0.013, OR = 2.152, 95% CI = 1.170–3.957) (Table 4).

## Discussion

### Discrepancies between North Korean medical journals and global standards

North Korean medical journals have unique characteristics distinguishing them from their global counterparts. Typically, medical articles adhere to the conventional introduction, methods, results, and discussion structure. However, articles in North Korean journals are organized around research objectives, subjects, methods, results, and conclusions, with the research objectives often substituting the introduction and being framed by directives from the ruling authority. This practice undermines the essential requirements of objectivity in academic articles. Such characteristics indicate that North Korean medical journals also serve as propagandistic publications, which is a common feature of North Korean publications in general.

Furthermore, the brevity of articles in North Korean journals—often less than two pages—limits the details and depth of the research presented. Such conciseness hinders the comprehensive evaluation of the quality of research, as there are typically insufficient details about their methods and inadequate discussion of the results. Notably, discussions of research ethics were absent, further reflecting the limitations of North Korean medical journals.

The predominant research areas in North Korean journals included pediatric gastroenterology and nutrition, allergy and respiratory diseases, surgery, cardiology, and neurology. By contrast, a study analyzing 201,141 pediatric-related articles indexed in MEDLINE/PubMed from 1998 to 2018 found that the most actively studied areas were epidemiology, neonatology, psychiatry, pediatric hematology-oncology, pediatric endocrinology, and pediatric cardiology, in that order [20]. This discrepancy suggests a distinct focus of North Korean research. Notably, epidemiological research was not found in North Korean medical journals, reflecting North Korea’s attitude toward not revealing health information to the outside world.

Most of the North Korean studies that were analyzed used case-control methods to examine the effects of drug therapies. Only a small fraction of studies employed other methods, with cohort studies accounting for only 0.2% of the sample and experimental studies accounting for 3.4%, while no randomized controlled trials were conducted. An evaluation of their research quality, based on SIGN, a global standard for evidence-based medicine, revealed that most studies were of low quality, failing to meet most criteria [18]. The quality assessment of research is crucial for establishing standards, evaluating evidence, and drawing robust conclusions. It is also instrumental for identifying flawed evidence and determining its applicability in clinical settings, which ultimately influences healthcare costs [21]. There is a notable high risk of bias across all areas in the analyzed North Korean research, including subject selection, confounding variables, intervention measurement, outcome evaluation, incomplete data, and selective reporting of results.

Given these issues, enhancing the methodological rigour and statistical power of North Korean studies is essential, particularly when applying the research findings to clinical practice. These limitations not only negatively affect subsequent research but also constrain the improvement of research capabilities in the field of pediatric science in North Korea.

### Classification of pediatric diseases and treatments

Based on the ICD-10, our analysis of the types of pediatric diseases reported in North Korean medical journals revealed that congenital malformations, infections, parasitic diseases, respiratory diseases, and gastrointestinal diseases were the most frequently examined topics. This distribution reflects the primary causes of pediatric mortality in North Korea and aligns closely with global trends observed in low- and middle-income countries. A systematic review and meta-analysis of observational studies from such countries, published between 2005 and 2021, identified infectious diseases, respiratory diseases, and gastrointestinal diseases as the leading causes of hospital mortality among children. Respiratory, infectious, and gastrointestinal diseases also ranked as the most common causes of pediatric hospital admissions [22]. The alignment between the focus of North Korean research and the global disease burden is further supported by a global study analyzing causes of death among individuals under 20 years of age across 204 countries from 1990 to 2021. That study highlighted neonatal diseases, lower respiratory infections, diarrhea, congenital malformations, malaria, and meningitis as leading contributors to disability-adjusted life years in low- and middle-sociodemographic index (SDI) countries [23]. Similarly, a WHO report analyzing pediatric mortality in North Korea from 2000 to 2015 corroborated these trends, emphasizing the significant role of respiratory infections, diarrhea, and congenital malformations in determining child health outcomes [19].

Over half of the analyzed research articles focused on diseases known to cause significant mortality, underscoring the strong correlation between research focus and the global burden of disease. This indicates that pediatric research in North Korea is strategically aligned to address the most burdensome diseases.

Significantly, 59.9% of the studies aimed to verify treatment efficacy. Unlike global trends in pediatric research that predominantly focus on epidemiology, North Korean pediatric studies mainly focus on treatments for diseases that impose significant burdens—in particular, studies related to pharmacotherapy accounted for 67.7% of the sample [20]. Traditional medicine (36.5%) was the most extensively studied field, followed by Western medicine (31.2%). Studies that used traditional medicine alone accounted for 27.3%, and those that employed integrative therapy (a combination of traditional and Western medicines) accounted for 9.2% of the sample.

North Korea has enacted legislation related to traditional medicine, stipulating under Article 7 of the Medical Law that the use of traditional medicine aims to improve the quality and standards of healthcare. It mandates that this be achieved through integration with Western medicine and explicitly requires the efficient application of traditional medicine in treatment [24]. This was also confirmed through the study objectives written in Kim Jong-il’s directives, illustrating the influence of North Korea’s policy directions on pediatric scientific research.

According to reports by defector physicians on North Korea’s healthcare system, traditional medicine began to develop asymmetrically owing to the lack of basic Western medicines during the Arduous March—the period of the North Korean famine and economic crisis—which lasted from 1996 to 1999. They noted that the destruction of pharmaceutical factories and the disappearance of drug trading made it more difficult to use Western medicine [25]. Additionally, this shortage led to the development of integrative therapy [26], which is a strategy for promoting self-reliance by expanding the use of traditional medicine based on the Juche ideology, North Korea’s national political and governing ideology. This integration played a critical role in addressing healthcare challenges at that time [27].

Extensive research related to traditional medicine has been conducted in the fields of gastroenterology, nutrition, and respiratory allergies. Studies of enteritis and asthma are particularly prevalent. Moreover, there has been greater emphasis on non-infectious and chronic diseases, compared with acute and infectious diseases. This is because chronic diseases allow for a safer, long-term evaluation of treatments’ effectiveness; unlike acute and infectious conditions, they do not require immediate therapeutic effects and seldom involve clearly identified causative agents.

Additionally, the research extended to nutritional supplements, which accounted for 9.2% of therapeutic studies. These studies explored the effects of nutritional supplements on malnutrition and gastroenteritis by comparing the efficacy of newly developed nutritional supplements in North Korea with that of existing supplements. This highlights the ongoing issues regarding pediatric nutrition in North Korea and suggests indigenous efforts to find solutions through research.

Furthermore, various other approaches—such as laser, hypothermia, light, and rehabilitation therapies—have also been studied, indicating that a diverse range of treatments beyond medication and surgery are being explored in North Korea. An article examining North Korea’s regulatory framework and policies concerning traditional medicine noted that the country emphasizes self-reliant solutions, aiming to address the inadequacy of pharmacological treatments by integrating non-pharmacological therapies [24]. Such research ultimately contributes to diversifying treatment methods and improving the quality of healthcare in North Korea.

### Changes in research trends over time

The volume of pediatric research articles in North Korea has gradually increased, signifying burgeoning interest and activity in pediatric science in the country. According to a study analyzing international scholarly articles authored by North Korean scientists from 2007 to 2016, international scholarly publications have also been increasing over time, with a notable increase since Kim Jong-un assumed power in 2012 [28]. This trend aligns with our findings, showing a higher growth rate of research articles during the Kim Jong-un era, compared with the Kim Jong-il era.

Differences in research focus between the two eras are evident. Under Kim Jong-il, the predominant research areas were gastroenterology and respiratory allergies. However, under Kim Jong-un, there has been a noticeable diversification and expansion of research fields, with a significant increase in surgical studies. This shift correlates with the policies of the Kim Jong-un regime, which emphasize science and technology and reflect concerted efforts toward national development. These policies have spurred active research in medical institutes and the establishment of large hospitals, suggesting a strategic enhancement of the healthcare infrastructure [29]. Based on such efforts, we can infer that there has been an increase in and diversification of pediatric research.

During the Kim Jong-il era, infectious and parasitic diseases dominated the research landscape. Under Kim Jong-un, however, the focus shifted toward congenital malformations and the emergence of endocrine, nutritional, and metabolic diseases as significant areas of study. These changes likely reflect improvements in the country’s economic conditions and overall health profile. Research comparing the causes of pediatric mortality in different countries shows that countries with a low SDI bear a higher burden of infectious diseases while the burden of congenital malformations increases as SDI levels rise [30]. This pattern suggests that North Korea saw improvements in economic conditions and living standards as the country transitioned from the Kim Jong-il era to the Kim Jong-un era. Given the increased food supply and improved dietary quality in North Korea during the 2010s, we can infer that the nutritional status of children has improved, leading to changes in disease patterns [31].

Significant differences in treatment were also observed between the ruling periods, with a higher frequency of traditional medicine use noted during the Kim Jong-il era, compared with the Kim Jong-un era, in studies of treatment efficacy. The Kim Jong-il regime emphasized the production of traditional medicines in the healthcare sector. The Kim Jong-un maintained a similar policy direction, aimed at integrating traditional and Western medicine, increasing traditional medicine production, and promoting the scientifization of traditional medicine, as found in research on the history of traditional medicine institutions and policies in North Korea [32]. However, there has been a notable decrease in interest in traditional medicine during the Kim Jong-un era, as evidenced by the absence of new initiatives or independent discourses related to it. This shift might be attributable to an increased emphasis on science and technology and a higher demand for informatization compared with the previous regime.

### Limitations of this study

First, as the literature review analyzes limited data, this study is inherently limited in its ability to generalize its findings. Additionally, achieving objectivity in analyzing North Korean medical research is challenging because of the propagandistic nature of North Korean publications, which often involve political interventions or publication biases.

Second, the qualitative evaluation results of the research articles analyzed in this study were low. The North Korean medical journals we examined often failed to meet the basic requirements mandated by international academic journals. Apart from the regularity of publication, there was also a lack of accurate information for proper evaluation [8]. Most articles were less than two pages, the research objectives often included the directives of North Korea’s leadership, and the articles did not describe specific statistical methods, presenting only simple results without CIs. There were no references to research ethics, and the number of references was limited to about six. Further, the articles did not mention biases or confounders and exhibited a high risk of bias in areas such as subject selection, outcome evaluation, incomplete evidence, and selective reporting of results. Such issues are compounded by North Korea’s closed environment, making practical verification and analysis difficult. Despite these challenges, given the extremely limited data available on North Korean medical research, such studies are still valuable for interpreting and indirectly inferring the status of pediatric research in North Korea.

## Conclusion

The North Korean pediatric research identified in this study was characterized by its limited yet specialized nature, coupled with its practical relevance. Moreover, there was an observable upward trend in pediatric research, with the spectrum of diseases studied evolving in response to economic progress and the increasing diversification of research and treatment modalities. Our findings demonstrate ongoing concerted efforts in the domain of pediatric medicine in North Korea toward indigenous medical advancement. Consequently, such initiatives could exert a positive influence on pediatric research in a future unified Korea.

## Data Availability

The data supporting this study’s findings are available from the corresponding author upon reasonable request.

## Abbreviations

CI: Confidence interval
SDI: sociodemographic index
SIGN: Scottish Intercollegiate Guidelines Network
UNICEF: United Nations Children’s Fund
WHO: World Health Organization

## References

[1] Daniel G, Loraine W, Peter J. A reassessment of mortality in North Korea, 1993–2008. Popul Div, U.S. Census Bureau; 2011.

[2] FAO, IFAD, UNICEF, WFP, WHO. The state of food security and nutrition in the world 2021. Rome: FAO; 2021.

[3] Christian P, Smith ER. Adolescent undernutrition: global burden, physiology, and nutritional risks. Ann Nutr Metab. 2018;72:316–28. 29730657 10.1159/000488865

[4] UNICEF Data. The state of the world’s children 2021. New York: UNICEF; 2021.

[5] Kim JE. Nutritional state of children in the Democratic People’s Republic of Korea (DPRK): based on the DPRK final report of the National nutrition survey 2012. Pediatr Gastroenterol Hepatol Nutr. 2014;17:135–9. 25349828 10.5223/pghn.2014.17.3.135 PMC4209317

[6] Lee SK. North Korean children: nutrition and growth. Ann Pediatr Endocrinol Metab. 2017;22:231–9. 29301183 10.6065/apem.2017.22.4.231 PMC5769832

[7] Kim KS, Lee CG. The National R & D system and S&T human resources training system in North Korea. Science and Technology Policy Institute; 2021.

[8] Ha S, Lee YH. Distribution of diseases studied in North Korean articles of ‘internal medicine’. Health Soc Welf Rev. 2018;38:589–610.

[9] Kim T, Choi S, Lee JY. Research topics in intellectual disabilities in North Korea: A scoping review. J Appl Res Intellect Disabil. 2022;35:374–81. 34704335 10.1111/jar.12956

[10] Kim HW, Jeon WT. Research output on mental health problems in North Korea between 2006 and 2017: A bibliographic analysis of North Korean medical journal articles. Asian J Psychiatr. 2020;53:102228. 32593086 10.1016/j.ajp.2020.102228

[11] Choi S, Kim T, Choi S, Shin HY. Surgical diseases in North Korea: an overview of North Korean medical journals. Int J Environ Res Public Health. 2020;17:9346. 33327471 10.3390/ijerph17249346 PMC7764982

[12] Lee CJ, Lee S, Kim HJ, Kang YA. Towards Understanding tuberculosis-related issues in North Korea: A narrative review of North Korean literature. Tuberc Respir Dis. 2020;83:201–10. 10.4046/trd.2019.0070 PMC7362750 32578408

[13] Ro DY, Kim DH, Park SH. North Korea must be global scale cohort, not a Galapagos in the medical research field. Asian Pac J Cancer Prev. 2019;20:2789–94. 31554378 10.31557/APJCP.2019.20.9.2789 PMC6976849

[14] Park DH, Choi MH, Lim AY, Shin HY. An analysis of infectious disease research trends in medical journals from North Korea. J Prev Med Public Health. 2018;51:109–20. 29631346 10.3961/jpmph.17.145 PMC5897231

[15] Arksey H, O’Malley L. Scoping studies: towards a methodological framework. Int J Soc Res Methodol. 2005;8:19–32.

[16] Levac D, Colquhoun H, O’Brien KK. Scoping studies: advancing the methodology. Implement Sci. 2010;5:69. 20854677 10.1186/1748-5908-5-69 PMC2954944

[17] The Joanna Briggs Institute. The Joanna Briggs Institute reviewers’ manual 2015 methodology for JBI scoping reviews. Adelaide: The Joanna Briggs Institute; 2015.

[18] Scottish Intercollegiate Guidelines Network. Methodology: checklists. Available from: https://www.sign.ac.uk/what-we-do/methodology/checklists/. Accessed Jun 24, 2024.

[19] World Health Organization. Global health estimates 2020: deaths by cause, age, sex, by country and by region, 2000–2019. Geneva: WHO; 2020.

[20] Levy-Mendelovich S, Barbash Y, Budnik I, et al. Pediatric literature trends: high-level analysis using text-mining. Pediatr Res. 2021;90:212–5. 33731817 10.1038/s41390-021-01415-8

[21] Lim SM, Shin ES, Lee SH, et al. Tools for assessing quality and risk of bias by levels of evidence. J Korean Med Assoc. 2011;54:346–55.

[22] Kortz TB, Mediratta RP, Smith AM, et al. Etiology of hospital mortality in children living in low- and middle-income countries: a systematic review and meta-analysis. Front Pediatr. 2024;12:1397232. 10.3389/fped.2024.1397232. 38910960 10.3389/fped.2024.1397232 PMC11190367

[23] GBD 2021 Causes of Death Collaborators. Global burden of 288 causes of death and life expectancy decomposition in 204 countries and territories and 811 subnational locations, 1990–2021: a systematic analysis for the global burden of disease study 2021. Lancet. 2024;403(10440):2100–32. 10.1016/S0140-6736(24)00367-2. 38582094 10.1016/S0140-6736(24)00367-2 PMC11126520

[24] Yi E, Kim D. Status of North Korea’s regulations and policy on Koryo medicine. Korean Herb Med Inf. 2021;9:95–108.

[25] Jeon WT. The future of unified healthcare. Pakyoungsa; 2023. pp. 45–91.

[26] Choi H. A comparison between physician training systems of South and North Koreas. PhD thesis, Seoul National University; 2020.

[27] Kim JS. A study on the characteristics and limitations of North Korea’s pharmaceutical policy. Health Soc Welf Rev. 2012;32:631–65.

[28] Noh KR, Kim EJ, Choi HK. A study on the production of science and technology knowledge in North Korea through international academic papers. J Korean Biblia Soc Libr Inf Sci. 2016;27:205–27.

[29] Shin H, Lee H, An K, Jeon J. North Korea’s trends on healthcare system in Kim Jong Un era: concentrated on healthcare delivery and organizational system. J Peace Unif Stud. 2016;8:181–211.

[30] GBD 2017 Child and Adolescent Health Collaborators. Diseases, injuries, and risk factors in child and adolescent health, 1990 to 2017: findings from the global burden of diseases, injuries, and risk factors 2017 study. JAMA Pediatr. 2019;173. 10.1001/jamapediatrics.2019.0337 PMC6547084 31034019

[31] Hong J, Park H, Park Y, Kim J, Jung E. North Korea in the Kim Jong-un era: a decade assessment and forecasts for 2022. Seoul: Korea Institute for National Unification; 2022.

[32] Kim D. The history of Koryo medicine system and policy in North Korea. Korean Herb Med Inf. 2021;9:45–56.

